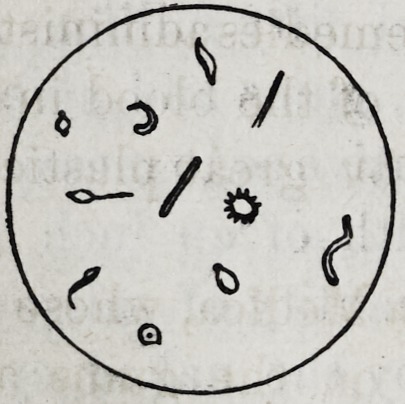# The Effect of Animalcules on the Teeth

**Published:** 1870-11

**Authors:** George B. Harrimore

**Affiliations:** Boston, Mass.


AMERICAN JOURNAL

OF

DENTAL SCIENCE.

Vol. IV. THIRD SERIES--NOVEMBER, 1870. No. 7.

### ARTICLE I.

### *The Effect of Animalcules on the Teeth.*

By George B. Harrimore, D.D.S., Boston, Mass.

Great enthusiasm in all that relates to dental science, now animates a very large number of dental practitioners. This is peculiarly gratifying as it will lead to further discoveries and bring to the dental art greater perfectness.

Already this enthusiasm has led to a variety of investigations, not only of matters directly in the line of dental science but of other subjects of remoter bearing, that every thing might be gathered which could enlarge the knowledge and skill of the dentist. With the continuance of this enthusiasm, the profession will be increasing both in power and usefulness.

The fact of the increasing number of dentists who are the most busy in the laboratory and in operating at the chair, and who yet find time for investigating the causes of injury to the teeth and of the best methods for the removal of these causes, argues well for future generations. Such investigations must result in discoveries, not only of violated physiological laws which have brought sad disasters to the mouth, but of other destructive agencies which have long been at work on the teeth without observation and hindrance.

We begin to experience the effects of these investigations. But for them the destructive power of living matter first known as *animalcules* and subsequently as infusoria, and more recently as *bioplasms*, would not be known. Having made this order of animal life a specialty in study, I am urged to submit to the public some of the results of my investigations.

The term animalcules, though not strictly connected with the nomenclature of modern zoology, is used as most familar to the ordinary mind, to denote some of the more minute forms of animal being for a knowledge of which we are indebted to the microscope.

Almost the first revelation made by this instrument was this extensive source of life. This was in the latter part of the last century when the microscope was in comparative rudeness as *Leuwenhoech* was experimenting with the instrument. *Gluchen* reduced this branch of life to a little system. He was succeeded by O. F. Müller who effected the first regular and scientific classification of animalcules. After this period the subject was at rest for some time and was at length revived by the celebrated Ehrenburg who published an elaborate work, and to whom more than to any other individual in former times we are indebted for definite knowledge on the subject. Since his publication the science has been prosecuted with vigor and success in France by Dujardin, in Germany by Tiebolet, Kolliker and others, in England by Owen and in the United States by Bailey.

The investigations of alternate generations by Stein and Agassez, have had the effect to stimulate other minds to research by the new light which these distinguished scholars and naturalists have thrown upon the subject.

As before intimated, by the earlier writers, animalcules covered all the life which was developed by the microscope. Plants and animals, mollusks, crustaceans, insects, worms and perfect forms were grouped together under the term "animalcules," and modern scientific men have bestowed great labor in their classification, and the result has been the formation of a class to which Müller gave the name Infusoria. An English writer, however, Lionel S. Beal, M. B. F. R. S., in a small, though elaborate and learned work lately published, gives to this class the name Bioplasm from the Greek words signifying life, and plasma. By way of explanation he says: "Now that the word Biology has come into common use it seems desirable to employ the same root in speaking of the matter which it is the main purpose of biology to investigate." It is of this order of life that I propose to treat in the present article, as it is found in almost every human mouth exerting a mighty power in the waste and consequent destruction of the teeth.

The salivary calculus which is precipitated in a greater or less quantity upon the natural or artificial teeth whenever the fluids of the mouth are at the best, is composed of earthy phosphates about eighty per cent. salivary mucus and flattened epithelium nearly twenty per cent. When this is subjected to the microscope and examined with one sixteenth of an inch immersion objective, and a high power eye piece or occular which magnifies about twenty-five hundred diameters, there can be seen living, moving bodies of divers forms and size, some long acicular, dish shaped, stellate and in a very few instances some bearing strong resemblance to the pimento berry, seemingly though not really more elongated.

My belief is that a cubic inch of tartar thus formed, is a deposit of no less than two hundred and fifty millions of this order of life existing about the teeth, gums and alveolar processes! These beings are so diminutive as to measure individually less than the fifty-thousandth of an inch in diameter. Wherever the most minute quantity of salivary calculus can be detected on the teeth by the naked eye, thousands of these busy beings may be witnessed by the aid of the proper magnifying power. As I have watched the activity of this industrious throng, and thought of spongy gums, absorbed alveolar process, and of the teeth quivering in the mouth, and furthermore as I have seen these bioplasms work their way to a single epithelium cell taken from the mucous membrane of the mouth and to all appearance, suck away a small portion of its contents, I have not hesitated long for the cause of this great disaster. These animalcules or bioplasms are continually at work, and at length rob the mouth which gives to the face its greatest beauty and expression. For the reason of their destructiveness in this regard, it is no marvel that, that noted man in dental practice and not less famed in dental science, Prof. W. H. Atkinson, of N. Y. City, reiterates to all in the profession "to clean the teeth is the first duty of the dentist to his patient; for cleanliness is irreseparable from health and beauty." The teeth, so necessary to the digestion of food, so instrumental in the manufacture of the atmosphere of the mouth, and so indispensible to a pleasing and attractive expression of countenance, should always be cleanly to be abiding and of a healthy tone. The dentist is to remember that not only are infusoria or bioplasms destructive to the gums, teeth and alveolar processes of the patient, but that they engender a fœtid breath of extreme unpleasantness to all coming in contact with it, and of great power in the production of bodily disease. He should, therefore, impress upon all under his care the necessity of having their mouths cleansed of these destructive agents.

The subjoined cut represents the smaller infusoria or bioplasms, not usually detected by a low power objective.

By this cut it will be seen that these little creatures are magnified to about twenty-five hundred diameters.

If the position taken in this article is correct, and I think it cannot be successfully controverted, some may inquire for a remedy. My treatment and the one I recommend is, in the first place, to cleanse the teeth of all extraneous substances, and then paint around the teeth and gums with a solution composed of one part by weight of crystalized carbolic acid and five of glycerine, to which two parts of water are added when it is ready for use.

It is a most singular phenomenon that the little creatures, infusoria or bioplasms, in their incredible numbers, their universal distribution, and their unsatiable voracity, devouring particles of matter which have decayed both animal and vegetable, for the making of the atmosphere we inhale salubrious, should in the human mouth become the ministers of disease and destruction. Nor can the phenomenon be solved on any other hypothesis than that the august Architect of the human frame, in his care for what appertains to the beauty and healthiness of man, designed that the mouth should have constant attention, that its fluids needful for health should be so treated as not to generate an order of life certain to work destruction to the teeth. Hence the lesson is most significant to the dentist and his patients. They are to heed this divine admonition and have the mouth free from these persistent little destroyers.

## Figures and Tables

**Figure f1:**