# Erratum. Dr. Geo. B. Harriman

**Published:** 1871-04

**Authors:** 


*Erratum.*—In the November number of the *Journal*, 1870, the name of the author of the article, entitled "The Effects of Animalcules on the Teeth," appears as Geo. B. Harrimore, D.D.S., which is an error, as it should be Geo. B. Harriman, D.D.S., Boston, Mass. In making out the Index of the present volume we discovered this error, and take the first opportunity offering to correct it.

